# The situational contingency of virtual and on-site workplace attendance decisions in Germany: a vignette-based approach

**DOI:** 10.1016/j.ssmph.2026.101979

**Published:** 2026-09-28

**Authors:** Joachim Gerich, Natalie Herdegen, Andreas Koch, Martin Kroczek, Philipp Kugler, Sophie-Charlotte Meyer, Mella Perleberg, Jochen Späth

**Affiliations:** a Department of Sociology Linz, Johannes Kepler University Linz, Linz, Austria; b Institute for Applied Economic Research at the University of Tübingen (IAW), Tübingen, Germany; c Federal Institute for Occupational Safety and Health (BAuA), Dortmund, Germany

**Keywords:** Sickness presenteeism, Sickness absence, Virtual sickness presenteeism, Factorial survey, Remote work, Occupational health

## Abstract

Sickness presenteeism (SP), i.e., working while ill, and sickness absence (SA) reflect alternative responses to ill-health. Although both stem from the same attendance decision process, little is known about how illness severity interacts with workplace conditions, how remote work opportunities influence attendance decisions and what role personal motives play. We draw on data from a factorial survey experiment following the 2024 BIBB/BAuA Employment Survey in Germany (N = 3532). Respondents evaluated ten hypothetical scenarios describing acute illness episodes that varied in symptom severity, infectiousness, workload, team conditions, attendance pressure, and weather. Multilevel logistic models estimate effects on the likelihood of working on-site, working remotely (if available), or calling in sick. Illness severity appears to be the main determinant of attendance behavior: increasing body temperature from normal to 38.3°C reduces the probability of working on-site by 54 percentage points among respondents without a remote work option. The option of working remotely is associated with more SP (64% vs. 55%) and a shift from on-site to virtual SP. Effects of contextual factors, such as attendance pressure, colleagues’ workload and infectiousness are smaller and vary with illness severity and work arrangement: they are most pronounced at moderate illness severity, while remote work can shift their influence from whether to how individuals work. Under severe illness, decisions to work are linked mainly to career- and reputation-related motives. Attendance decisions thus seem to follow a hierarchical logic with illness severity as the primary determinant and other factors depending on illness severity and work arrangements.

## Highlights

• Illness severity is the key driver of health-related attendance decisions
• Situational factors matter mainly under mild or moderate illness
• Remote work raises the illness severity threshold for taking sick leave
• Under severe illness, attendance decisions reflect personal motives

## 1 Introduction

While there is a long history of research on sickness absence (SA), research on sickness presenteeism (SP), defined as working in the state of ill-health (Ruhle et al., 2019), has only gained broader attention in recent decades. This seems surprising as both SA and SP can be seen as outcomes of the same underlying decision-making process of workers facing health complaints. Following the conceptual presenteeism model of Johns (2010) the regular attendance of workers is disrupted when individuals face an illness which may be of an acute, chronic or episodic nature. In light of the nature and severity of the health event (i.e., the extent to which it limits work-ability), employees have to decide between two options, that is to call in sick or attend work despite illness.

This decision is not only widespread but also consequential for individuals and organizations. Previous research has predominantly focused on negative outcomes of SP and confirmed detrimental effects on health (Demerouti et al., 2009; Kivimäki et al., 2005; Skagen & Collins, 2016; Taloyan et al., 2012) as well as productivity and costs (Goetzel et al., 2004; Miraglia & Kinman, 2017; Stewart et al., 2003). Recently, some studies also highlight potential personal benefits such as avoiding work pile-up, maintaining relationships, career prospects or perceived self-efficacy (Biron et al., 2022; Karanika-Murray & Biron, 2020; M. Wang et al., 2022; Y. Wang et al., 2023). SP is therefore both a health-relevant and strategic behavior embedded in complex trade-offs.

Attendance decisions involve weighing anticipated consequences for one's own health, performance, reputation, and feelings of guilt, as well as implications for others (e.g., colleagues or clients). Moreover, these decisions depend on personal constraints (e.g., work attitudes, family demands, stress) and context-related conditions such as actual workload, ease of replacement, leadership styles, job security or organizational attendance pressures (Aronsson & Gustafsson, 2005; Biron & Saksvik, 2009; Johns, 2010; Lohaus & Habermann, 2019). Hence, an individual's decision between SA and SP is likely contingent on various situational boundary conditions, in which employees seek an “equilibrium” between different anticipated outcomes (Luksyte et al., 2023; Ruhle et al., 2019; Whysall et al., 2018). However, despite well-developed conceptual models of attendance decision-making, empirical evidence for these situational contingencies remains scarce.

The increased availability of flexible work arrangements such as working remotely, accelerated by the COVID-19 pandemic, adds further complexity to attendance decision-making. Instead of calling in sick or working on-site despite sickness, employees may work remotely while ill, denoted as “virtual presenteeism” or “workahomeism” (Brosi & Gerpott, 2023; Fiorini, 2024; Ruhle & Schmoll, 2021). Remote work may enable SP by eliminating commuting, allowing individualized work arrangements, or enabling reduced work effort. Furthermore, concerning infectious disease, virtual SP eliminates the risk of transmission. Therefore, Fiorini (2024) argues that the option of virtual SP could raise the threshold of sickness severity at which employees perceive illness as justifying sick leave.

Previous studies on attendance behavior have relied largely on retrospective survey data (e.g., SA and SP days during the past year), which limit the ability to capture situational variation, are prone to recall bias, and conflate decision-making with underlying health vulnerability (Gerich, 2015). Experimental approaches are therefore needed to isolate the causal effects of situational factors. Against this background, the present study uses a factorial survey experiment to examine the role of illness severity, workplace conditions, remote work opportunities, and personal motives in work attendance decisions.

## 2 Theoretical background

Previous research has identified a large number of personal, task-related, and organizational characteristics related to SP behavior (Lohaus & Habermann, 2019; Miraglia & Johns, 2016; Ruhle et al., 2019). Despite strong evidence that these factors may affect attendance decisions, ill-health has to be considered as a central “gatekeeper” (Steers & Rhodes, 1978) of attendance behavior. On the one hand, as already assumed by others (Halbesleben et al., 2014; Johns, 2010), attending work – whether on-site or at home – can be expected as the default behavior of healthy individuals. On the other hand, severe illness will impair employees’ ability to work and therefore SA can be expected as the default decision of those faced with severe symptoms. However, situations of mild or moderate symptoms will be associated with the highest ambiguity related to the decision-making process. Consequently, Steers and Rhodes (1978) formalized work attendance as a function of motivation and ability to attend, where motivation influences attendance only if the employee has the ability to attend work. In their model, motivation consists of three key components: (1) affective response to the job situation (e.g., job stress, team cohesion) (2) internal pressure (e.g., work ethic, loyalty, commitment), and (3) external pressures (e.g., managerial attendance expectations, workplace norms). Similarly, a model proposed by Whysall et al. (2023) suggests that attendance decisions unfold through a series of hierarchical steps. In a first stage, individuals assess the extent to which their symptoms impair their ability to work. Other factors, such as workload, organizational absence policies, or preferences and values, are typically considered in subsequent stages of the decision-making process. Drawing on the results from a qualitative diary study, H. Chen (2024) concludes that individuals tend to “skip” these later stages when experiencing either very mild or very severe symptoms, where SP and SA seem to be the default choices irrespective of other boundary/contextual conditions. Hence, we expect symptom severity to be the strongest predictor of attendance decisions. We further expect that the effects of other dimensions on attendance decisions (such as workload, team cohesion, or managerial attendance pressure) will be more pronounced in situations of moderate symptom severity but less relevant when symptoms are either very mild or very severe. Moreover, since virtual SP may raise the threshold of illness severity that justifies SA (Fiorini, 2024), we expect that other contextual factors may still be relevant in attendance decisions even at higher levels of illness severity, when individuals have the option of working remotely. However, although we expect SA as the default decision for most individuals facing strong symptoms, some employees might still choose SP despite more severe illness. We assume that this may occur when individuals prioritize other values and motives above considerations regarding their health. Therefore, we expect that those who opt for SP in situations where they are faced with more severe symptoms, more frequently report internal SP motives such as work ethic, avoidance of negative consequences due to SA, maintenance of their organizational reputation or the well-being of others.

### 2.1 Vignette dimensions: expected factors shaping work attendance decisions

Building on the attendance behavior model by Steers and Rhodes (1978) and, in part, the presenteeism decision model by Whysall et al. (2023), we distinguish ability and motivation as key determinants of work attendance, each shaped by distinct factors. Ability refers to physical or external conditions that make attendance feasible, whereas motivation encompasses individual and contextual drivers that make attendance desirable or expected. We structured our vignette dimensions accordingly: symptom and illness severity capture physical capacity as a fundamental prerequisite; infectiousness is treated as a specific constraint, especially salient in team-based settings due to transmission concerns. Beyond ability, both frameworks emphasize motivational influences. Steers and Rhodes highlight internal and external pressures, while Whysall et al. articulate how personal values, workplace expectations, and anticipated outcomes shape decisions in later stages. Guided by these models, we include the following contextual dimensions reflecting motivational factors, i.e., individual workload, team workload, team cohesion, and attendance pressure, as well as external factors such as weekday and weather as vignette dimensions in our factorial survey experiment.

#### 2.1.1 Sickness characteristics

To model health-related impairment in a way that respondents can easily relate to, we vary common cold symptoms (i.e., feeling weak and exhausted, having a headache) as well as body temperature. We deliberately focused on acute, short-term symptoms because they are widely recognizable, can be varied systematically in hypothetical scenarios, and typically require an immediate attendance decision. Moreover, indicators of infectiousness were included as a distinct aspect of illness severity, as they may play a critical role in attendance decisions, especially with regard to on-site work, given the potential risk of contagion.

#### 2.1.2 Workload

The workload employees face can substantially shape their motivational decisions regarding attendance (J. W. Chen et al., 2023; Deery et al., 2014; Demerouti et al., 2009; Y. Wang et al., 2023). When employees perceive an overwhelming backlog of tasks, they may feel compelled to attend work despite illness, in order to avoid a pile-up of work and meet expectations. Rivkin et al. (2022) highlight that employees use SP as a coping strategy when work demands are high.

#### 2.1.3 Team workload

When directly asked about their motivations for going to work despite feeling unwell, employees often state that they do not like letting their colleagues down, as their absence would create an additional workload for others (Baker-McClearn et al., 2010; Collins & Cartwright, 2012; Grinyer & Singleton, 2000). Therefore, we expect the probability of choosing SP to increase in situations where an individual's absence would impose a higher workload on colleagues.

#### 2.1.4 Team cohesion

Strong team cohesion has been shown to encourage SP due to a desire to support the group and maintain harmony. Research by M. Wang et al. (2022) suggests that employees in cohesive teams are more likely to prioritize the team's performance over their own health, potentially leading to increased SP. In such contexts, employees may be motivated by a desire to maintain positive team dynamics and support collective success, thus engaging in voluntary SP (Van Waeyenberg, 2023). Conversely, low team cohesion may discourage proactive behaviors (e.g., job crafting) and reduce the likelihood of working while ill (M. Wang et al., 2022), although the evidence is mixed. Miraglia and Johns (2016) found a weak negative association between collegial support and SP in their meta-analysis, suggesting that supportive environments may facilitate illness disclosure and normalize SA rather than SP.

#### 2.1.5 Attendance pressure

Attendance pressure, rooted in organizational culture, substantially influences employees' attendance behavior. In organizations with strong attendance norms, employees are more likely to engage in SP, driven by fear of repercussions and workplace social climate (Mach et al., 2018; Ruhle & Süß, 2020). This pressure limits employees' freedom to call in sick (Biron & Saksvik, 2009).

#### 2.1.6 Weekday

Research on SA shows unequal distributions across weekdays, with higher absence rates on Mondays compared to later in the week (Barmby, 2002; Bryson & Forth, 2007; Butler et al., 2014). This “Monday effect” is often linked to shirking behavior or low motivation (Libet et al., 2001), but may also partly reflect illnesses that begin during the weekend and are first reported on Monday (Barmby, 2002). However, most studies focus on SA and overlook SP. An alternative explanation is that employees who are unwell may choose SP later in the week, in anticipation of recovering over the weekend.

#### 2.1.7 Weather

Weather conditions can affect attendance behavior through ability- and motivation-related factors. Severe weather can hinder on-site attendance due to commuting challenges, though remote work options may reduce this barrier. Favorable weather, however, may lower motivation to attend as employees prioritize leisure (Coleman & Schaefer, 1990; Shi & Skuterud, 2015). Unfavorable weather can also dampen mood and intrinsic motivation, especially for those with health issues and may discourage commuting due to concerns about worsening the physical condition. Additionally, attending work while ill requires higher motivation, which bad weather can diminish, making employees more likely to stay home when unwell.

### 2.2 Study objectives and research questions

Taken together, the preceding theoretical arguments suggest that illness severity is likely to be the primary determinant of attendance decisions, whereas other contextual boundary conditions become particularly relevant when attendance decisions are ambiguous. The availability of remote work may further modify this decision process by providing an additional attendance option. To examine these expectations, we employ a factorial survey experiment (Auspurg & Hinz, 2015) in which respondents evaluated ten systematically varied scenarios of an acute health complaint and indicated whether they would call in sick, attend work on-site, or work remotely. This design enables causal inference regarding the effects of situational characteristics on attendance decisions (see Gerich et al., 2025).

Specifically, we address the following research questions: (1) How does illness severity, interacting with illness- and work-related situational characteristics, influence employees' attendance decisions when facing acute health complaints? (2) How does the availability of remote work affect these decisions and the relative importance of situational factors? (3) Which motives underlie employees’ decisions to work while ill, and how do these motives vary with illness severity and work context?

## 3 Data and methods

### 3.1 Data

The data originate from a factorial survey experiment on SP behavior, implemented as a follow-up to the BIBB/BAuA Employment Survey 2024, a nationally representative telephone survey of approximately 20,000 employed individuals in Germany (Gensicke et al., 2024). The gross sample for the follow-up survey comprised 15,074 dependent employees aged 18 or older who had participated in the preceding BIBB/BAuA Employment Survey and had agreed to be recontacted. All individuals in this gross sample were invited to participate in the factorial survey, which was conducted as a computer-assisted web interview (CAWI) in 2024, resulting in 5106 completed interviews. Respondents were randomly assigned to one of several vignette-based experiments, with the majority (n = 3585) being allocated to the main experiment described here (for details, see Gerich et al., 2025). The present study therefore includes respondents assigned to this main experiment. Each participant evaluated 10 hypothetical scenarios, which were presented sequentially in the online questionnaire. In each scenario, respondents woke up feeling unwell and had to decide whether to go to work, stay home without working, or work remotely (if applicable). The vignettes systematically varied in symptoms, job characteristics, and contextual factors. A randomly selected subsample of 520 of these respondents in the main experiment received additional health information on SP as part of an embedded information experiment.^1^ Assignment to the corresponding experimental conditions was based on gender, age, and whether respondents worked in a project-based environment. Because these variables were required to account for experimental-group assignment in the analyses, respondents with missing information on any of them were excluded. After applying these restrictions, the final analytical sample comprised 3532 individuals.

### 3.2 Measures

The following text presents an example vignette, in which underlined elements indicate the systematically varied dimensions (labels of vignette dimensions in square brackets were not shown to respondents, see also Table A1 in the Appendix).

“*You wake up on a Monday [weekday] morning. It’s bright outside, and the sun is shining into your bedroom [weather]. As you get up, you realize that you’re not feeling well. You feel weak and exhausted, and you have a mild headache [cold symptoms]. Your body temperature is slightly elevated (37.5°C) [body temperature]. In the past few days, you have not been in contact with anyone showing similar symptoms [infectiousness]. Your supervisor has recently emphasized that taking sick leave due to illness is completely acceptable [attendance pressure]. You are currently working with people you enjoy working with [team cohesion]. Your personal workload is currently higher than usual [workload]. You expect that your colleagues will face significantly more work [team workload] if you don’t show up.”*

The primary outcome variable was the self-reported attendance decision in each vignette, measured as a categorical variable (“Given these circumstances, would you …”) with the options “not work” (*SA*), “go to work (on-site)” (*on-site SP*), and “work remotely” (*virtual SP*). The virtual SP option was only offered to respondents who had indicated in a previous question that they have the option of working remotely.

The following vignette dimensions were randomly varied (see Appendix for the exact wording): Weekday (Monday to Friday), weather (good/bad), cold symptoms (weak/medium/strong), body temperature (normal/37.5°C/38.3°C), infectiousness (infectious/not infectious), attendance pressure (no/low/high pressure), team cohesion (low/high), personal workload (low/normal/high), team workload (colleagues’ workload affected/not affected).

All vignette dimensions were varied orthogonally using a D-efficient design, ensuring both statistical identification of main and selected interaction effects and reasonable independence across dimensions (D-efficiency = 95.58; all intercorrelations r ≤ 0.03). The levels are evenly distributed within the dimensions (see Table A2 in the Appendix).

Respondents who opted for on-site or virtual SP in the last vignette were asked to rate their motives for doing so based on seven items using a five-point scale ranging from ‘very unimportant’ to ‘very important’. The items reflect both approach- and avoidance-oriented motives to work despite illness (Ma et al., 2018; Van Waeyenberg, 2023; Y. Wang et al., 2018):

• Personal principles and inner beliefs (“My inner convictions and principles”)
• Social evaluation (“What colleagues or supervisors would think of me”)
• Reputation within the organization (“My reputation within the company”)
• Career and professional advancement (“My professional success and career”)
• Avoidance of inconvenience (“Avoiding inconvenience”)
• Concern for others' wellbeing (“The well-being of others (e.g., customers, clients)")
• Pile-up of work (“That unfinished work piles up”)

### 3.3 Analytic strategy

To analyze the effects of the vignette dimensions on work attendance decisions, we employed multilevel regression models that account for the nested structure of the data. All models were estimated using random intercept regression models to account for intra-individual correlation. In a first step, we examined the main effects of the vignette dimensions on attendance behavior. For respondents without remote work option, we estimated binary logistic regression models (on-site SP vs. SA). For those respondents with remote work option, we used multinomial logistic regression models, distinguishing between three response categories (SA, on-site SP, virtual SP). To account for the fact that certain groups of respondents were less likely to participate in the main experiment due to their allocation to another experiment, we included information of whether respondents were potentially qualified for an assignment to another sub-experiment as a control variable. As the vignettes were randomly assigned to respondents, vignette characteristics are, by design, independent of individual characteristics. Additional individual-level covariates are therefore not required to identify the effects of the experimentally varied vignette dimensions. However, respondent characteristics may still be relevant for the generalizability of the findings and for potential heterogeneity in attendance decisions.^2^ To facilitate interpretation and comparability across models and variables, we report Average Marginal Effects (AME), which quantify the average effect of a vignette characteristic on the predicted probability of an outcome. In a second step, we included interaction terms between body temperature and the other vignette dimensions to analyze the conditional relevance of contextual factors across levels of symptom severity. Finally, we analyze the motives underlying work attendance decisions, using linear regression models and regress the different motives on the body temperature of the final vignette. Additional vignette characteristics and an indicator for experimental group membership are included as controls. Separate models are estimated for virtual and on-site SP, allowing for a differentiated view of motivational patterns across the two attendance types.

## 4 Results

Table 1 summarizes the characteristics of the sample and the descriptive statistics of the SP motive items. The analytical sample covers employees from a broad range of occupational backgrounds, with primary and secondary service occupations clearly dominating. Respondents are 52 years old on average, 41% are female, and 58% report having at least some opportunity to work remotely. Just under two-thirds (65%) of respondents work full-time, 70% hold a vocational degree, and 38% hold an academic degree. Regarding school education, 62% completed upper secondary education, 28% completed intermediate secondary education, and just under 10% completed lower secondary education.

**Table 1:** Summary statistics.

	Mean	SD	N (Respondents)
**Individual characteristics**			
Age in years	52.341	10.277	3560
Female	0.408	0.492	3573
Working (sometimes) remotely	0.577	0.494	3573
Contractual weekly working hours ≤35	0.346	0.009	3095
Contractual weekly working hours >35	0.654	0.009	3095
*Occupational categories*^a^			
Production-related occupations	0.116	0.005	3538
Primary service occupations	0.383	0.008	3538
Secondary service occupations	0.501	0.008	3538
*Vocational/academic qualification*			
Vocational degree	0.700	0.008	3521
Academic degree	0.379	0.008	3521
No degree	0.026	0.003	3521
*School attainment*			
Lower secondary education	0.096	0.005	3570
Intermediate secondary education	0.278	0.008	3570
Upper secondary education	0.622	0.008	3570
Other school degree	0.004	0.001	3570
**Motives to work despite illness**^b^ (*1: very unimportant to 5: very important)*			
Inner convictions and principles	4.287	0.803	2078
What colleagues or supervisors think of me	2.485	1.060	2079
Reputation in the company	2.622	1.079	2069
Professional success and career	2.318	1.024	2069
Avoidance of unpleasantness	2.677	1.152	2069
Well-being of others	3.728	1.000	2051
Accumulation of unfinished work	3.283	1.140	2052

Notes:

a Occupational categories: production-related activities: physical tasks related to extraction, manufacturing, construction, plant operation, and equipment repair; primary service activities: routine services that directly support the production flow or enter final consumption (e.g., transport, trade, standard clerical work); secondary service activities: intangible, knowledge-intensive tasks (“knowledge work”) that qualitatively enhance production (e.g., R&D, management, consulting, and education) (Tessaring, 1994).

b Restricted to employees choosing SP in the last vignette.

### 4.1 Main effects of situational factors on attendance decisions

Table 2 shows the AMEs of the vignette characteristics on respondents’ work attendance decisions. Column (1) refers to individuals without a remote work option (binary logit model on-site SP vs. SA). Columns (2)-(4) refer to individuals with a remote work option based on a multinomial logit model.

**Table 2:** Average Marginal Effects (AMEs) of vignette characteristics on work attendance.

	Respondents without remote work option	Respondents with remote work option			Pseudo-R^2^	
	On-site work	Not working	Remote work	On-site work	Without remote work option	With remote work option
	(1)	(2)	(3)	(4)	(5)	(6)
**Day**					0	0
Monday (base)						
Tuesday	−0.010	0.006	0.003	−0.008		
	[-0.025, 0.005]	[-0.007, 0.019]	[-0.011, 0.016]	[-0.018, 0.001]		
Wednesday	−0.002	0.001	0.001	−0.002		
	[-0.018, 0.013]	[-0.011, 0.013]	[-0.012, 0.014]	[-0.012, 0.007]		
Thursday	0.001	−0.002	0.006	−0.005		
	[-0.014, 0.016]	[-0.015, 0.011]	[-0.008, 0.020]	[-0.015, 0.005]		
Friday	0.008	−0.002	0.008	−0.006		
	[-0.007, 0.023]	[-0.015, 0.010]	[-0.005, 0.022]	[-0.015, 0.004]		
**Weather**					0	0
Good weather (base)						
Rainy weather	0.001	**0.011**	−0.002	**−0.009**		
	[-0.008, 0.011]	[0.003, 0.019]	[-0.010, 0.006]	[-0.015, −0.003]		
**Cold symptoms**					0.039	0.033
Weak symptoms (base)						
Medium symptoms	−0.005	−0.004	0.005	−0.002		
	[-0.016, 0.006]	[-0.013, 0.006]	[-0.006, 0.016]	[-0.010, 0.006]		
Strong symptoms	**−0.119**	**0.121**	**−0.064**	**−0.057**		
	[-0.133, −0.104]	[0.109, 0.134]	[-0.077, −0.051]	[-0.066, −0.049]		
**Body temperature**					0.325	0.245
Normal (base)						
37.5°C	**−0.153**	**0.086**	**0.025**	**−0.111**		
	[-0.170, −0.135]	[0.074, 0.098]	[0.009, 0.041]	[-0.126, −0.096]		
38.3°C	**−0.537**	**0.471**	**−0.191**	**−0.280**		
	[-0.560, −0.514]	[0.452, 0.490]	[-0.211, −0.171]	[-0.298, −0.263]		
**Infectiousness**					0.002	0.002
Infectious (base)						
Not infectious	**0.027**	**−0.015**	−0.006	**0.021**		
	[0.017, 0.037]	[-0.023, −0.007]	[-0.015, 0.002]	[0.015, 0.027]		
**Pressure**					0.001	0.001
No pressure (base)						
Low pressure	0.015	−0.013	0.006	0.007		
	[0.003, 0.027]	[-0.023, −0.003]	[-0.005, 0.016]	[0.000, 0.014]		
High pressure	**0.020**	−0.008	−0.003	**0.012**		
	[0.007, 0.032]	[-0.018, 0.001]	[-0.014, 0.008]	[0.004, 0.019]		
**Team cohesion**					0.001	0.001
Like team (base)						
Not like team	**−0.014**	**0.021**	−0.007	**−0.014**		
	[-0.023, −0.004]	[0.013, 0.029]	[-0.016, 0.002]	[-0.020, −0.008]		
**Workload**					0	0
High workload (base)						
Normal workload	0.001	0.007	−0.010	0.003		
	[-0.011, 0.013]	[-0.003, 0.018]	[-0.021, 0.001]	[-0.005, 0.010]		
Low workload	−0.005	**0.017**	**−0.018**	0.001		
	[-0.018, 0.007]	[0.007, 0.027]	[-0.028, −0.007]	[-0.007, 0.008]		
**Teamload**					0.008	0.004
Colleagues affected (base)						
Colleagues not affected	**−0.047**	**0.038**	**−0.023**	**−0.015**		
	[-0.058, −0.037]	[0.029, 0.047]	[-0.033, −0.014]	[-0.021, −0.009]		
Control for potential participation in other experiment	✓	✓	✓	✓		
N	14,870	20,450	20,450	20,450		
Overall Pseudo R^2^					0.425	0.402

*Note:* Average marginal effects from binary (Column 1) and multinomial (Column 2-4) multilevel regressions; Values in brackets represent 95% confidence intervals; Bold values indicate statistically significant coefficients (p < 0.05); Standard errors clustered at the respondent level.

#### 4.1.1 Sickness characteristics

The results show that the sickness characteristics have the highest impact on work attendance decisions. Among all factors, body temperature has the most substantial individual effect on work attendance. At a moderately elevated body temperature (37.5°C), the probability of attending work on-site decreases by 15 percentage points relative to the baseline of normal body temperature. At a high body temperature (38.3°C), the probability declines sharply by 54 percentage points. The availability of a remote work option markedly alters the decision-making situation. While the likelihood of SA still increases substantially with a high body temperature (38.3°C) by 47 percentage points, it rises only moderately by around 9 percentage points at 37.5°C. However, the tendency for virtual SP significantly increases at 37.5°C but significantly declines again at 38.3°C**.**

Despite the strong negative impact of (high) body temperature on the decision to work, the average propensity for on-site and virtual SP remains above zero. Table 3 displays the relative frequency of different attendance decisions by body temperature. At a body temperature of 38.3°C, respondents without a remote work option choose SA in 76 percent of the cases (i.e., they choose SP 24 percent of the cases). Those with access to remote work opt for SA in 66 percent, decide to work from home in 28 percent, and still attend the workplace in 7 percent of the scenarios. When collapsing virtual and on-site SP into a single category, the overall propensity of SP at a body temperature of 38.3°C is 30 percent in the total sample (Column 5, Table 3). In general, this pattern holds for both genders, with modest differences (see Table A3 and A4 in the Appendix). Men are slightly more likely to work while ill: at 38.3°C, choosing presenteeism in 32 percent of cases compared with 28 percent of cases for women. With a remote work option, men are more inclined to show virtual SP, whereas women more often choose to work on-site. Without a remote work option, men also show higher on-site SP (26 versus 21 percent).

**Table 3:** Relative frequency of different choices.

	Respondents without remote work option	Respondents with remote work option			All respondents
	Working	Not working	Remote work	On-site work	Working^a^
	(1)	(2)	(3)	(4)	(5)
**Body temperature**					
Normal	0.799	0.155	0.450	0.394	0.825
37.5°C	0.627	0.257	0.505	0.237	0.694
38.3°C	0.237	0.655	0.277	0.068	0.300
N	14,870	20,450	20,450	20,450	35,320

*Note*:

a Total sample without differentiation if remote work is possible or not.

With regard to the cold symptoms, we find significant and sizeable effects only for strong symptoms (i.e., feeling weak, exhausted with severe headache). In this case, the likelihood of choosing to work decreases by 12 percentage points relative to weak symptoms in the binary case. In the multinomial specification, the propensity of not working increases by 12 percentage points, while the probabilities of remote work and on-site work both decrease by around 6 percentage points (Table 2). As expected, the potential infectiousness reduces the decision to opt for on-site SP, but not for virtual SP.

#### 4.1.2 Work-related factors

Work-related factors also significantly influence the decision-making behavior. The propensity for SA decreases, and the propensity for on-site SP increases with higher attendance pressure. However, the propensity for virtual SP remains unaffected. The respondent's personal workload increases only the likelihood to opt for virtual SP instead of SA. Colleagues' workload plays a more substantial role in the decision to work while ill. The respondents are more inclined to virtual and on-site SP when their absence would raise their colleagues' workload. Team cohesion also influences the decision-making behavior. In scenarios, in which respondents have to work with colleagues they do not get along with, the probability of choosing SA increases and the probability for on-site SP decreases. Differentiating between remote work and on-site work, we find that the likelihood of on-site SP decreases in these situations, while the tendency to work remotely remains unaffected.

#### 4.1.3 External factors

The external factors in our factorial survey setup exert much less influence on attendance decisions. The weekday has no impact, while weather conditions do affect attendance, but only for respondents with a remote work option. For this group, bad weather conditions decrease the probability of on-site SP and increase the probability of SA. Somewhat surprisingly, the decision for virtual SP remains unaffected, indicating that in bad weather, respondents are more likely to refrain from working at all, rather than substituting commuting with remote work.

The explanatory contribution of each of the different vignette dimensions indicated by Pseudo-R^2^ values (Table 2, Columns 5 and 6) confirms that sickness characteristics, and body temperature in particular, account for the largest share of variance explained. While infectiousness and work-related characteristics also significantly affect attendance decisions, their relative explanatory contribution is considerably smaller.

### 4.2 Interaction models:conditional effects of vignette dimensions by body temperature

Fig. 1 displays the predicted probabilities of working on-site (versus not working) among respondents without a remote work option in a margins plot, based on a binary logistic regression model that interacts body temperature with all vignette dimensions (see also Table A5 in the Appendix). The circles, diamonds, and triangles represent the predicted probabilities of working on-site at a normal body temperature, at 37.5°C, and at 38.3°C body temperature, respectively. Error bars indicate 95% confidence intervals, and vertical lines mark the mean predicted probability of working at each body temperature level. Consistent with the results presented in Table 2, the predicted probability of working on-site is lowest across all levels of the other vignette dimensions at a body temperature of 38.3°C, followed by 37.5°C and highest at normal body temperature. This pattern reflects the clear severity gradient in the binary attendance decision. While the overall decline in on-site work with increasing body temperature is robust across vignette dimensions, notable variations emerge for specific factors. In particular, cold symptoms, infectiousness, effects on colleagues' workload, and attendance pressure show stronger conditional effects. These differences are especially pronounced at moderately elevated temperatures (37.5°C) for the dimensions of attendance pressure and, to a lesser extent, for infectiousness and effects on colleagues’ workload, suggesting that these contextual factors exert greater influence when the decision to attend work on-site is less clear-cut. The severity of cold symptoms affects attendance decisions more strongly at normal or slightly elevated compared to high body temperature.

**Fig. 1:**
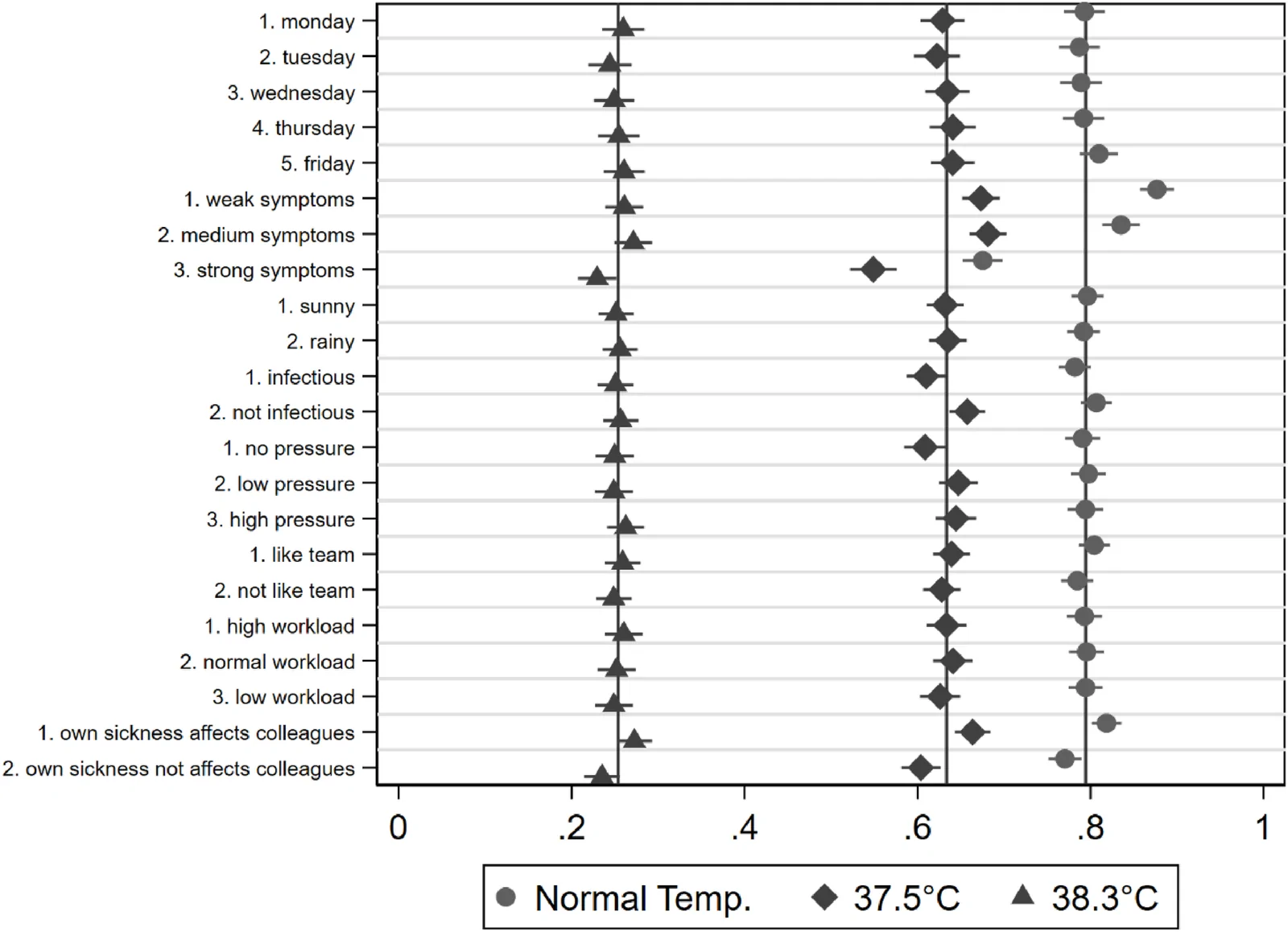
Predicted probabilities of working on-site by body temperature across vignette dimensions among respondents without a remote work option *Note:* Results are based on a binary logistic regression model including interactions between body temperature and all vignette dimensions, see also Table A5 in the Appendix. *Source*: Factorial survey on sickness presenteeism conducted as a follow-up to the BIBB/BAuA Employment Survey 2024.

While the margins plot provides a clear and intuitive illustration of how body temperature moderates contextual effects in a binary decision context, the decision structure becomes more complex once the option of remote work is introduced, as contextual factors may affect not only *whether* individuals work or not, but also *how* they work. We therefore refrain from plotting the multinomial interactions. Instead, Table A6–A8 in the Appendix report average marginal effects from the same multinomial logistic regression model with interactions between body temperature and all vignette dimensions, shown separately by outcome category. 3

Despite this added complexity, several vignette dimensions continue to exhibit interaction patterns that are largely quasi-binary in nature, such as weather conditions, cold symptoms and infectiousness. For example, rainy weather significantly reduces the probability of on-site SP at normal body temperature (AME = −0.020, Table A7) while significantly increasing the likelihood of SA (AME = 0.018, Table A8), with significantly weaker effects at higher body temperature levels. Similarly, strong cold symptoms decrease on-site SP (AME = −0.115, Table A7) and virtual SP (AME = −0.074, Table A6) and substantially increase the probability of SA (AME = 0.188, Table A8) at normal temperature, but these effects are also attenuated at higher temperature levels. In contrast, team cohesion and attendance pressure operate in a genuinely multinomial way. Here, body temperature not only affects the strength of these effects, but also changes what kind of decision individuals are facing, thus primarily structuring how work is carried out rather than whether work takes place at all. Attendance pressure is associated with a relocation of virtual SP (AME = −0.035, Table A6) to on-site SP (AME = 0.026, Table A7) at normal body temperature, whereas the probability for SA remains at a low level. Lower team cohesion is associated with a higher probability of SA at a normal (AME = 0.030, Table A8) and at a high body temperature (AME = 0.028, Table A8). However, whereas at a normal body temperature this increase in SA is mainly substituted by on-site SP (AME = −0.021, Table A7), it is substituted by virtual SP at a high body temperature (AME = −0.019, Table A6).

Taken together, the multinomial interaction results indicate that body temperature moderates contextual effects in two different ways. For some dimensions, increasing severity mainly strengthens or weakens a largely binary decision about attending work. For others, such as team cohesion and attendance pressure, symptom severity changes the nature of the decision itself, shifting contextual influence from whether individuals work to how work is organized (on-site vs. remote), as long as working remains feasible.

### 4.3 Motives for working while ill

In a final step, we assess how respondents rate the importance of different motives for showing SP and whether the importance varies with body temperature. Specifically, we estimate separate linear regression models for each of the seven motives reported after the final vignette, treating the motive ratings as dependent variables and body temperature as the key explanatory variable. As shown in Table 4, higher body temperature is positively associated with the reported importance of several motives for working while ill. As illness severity (i.e., body temperature) increases, employees who still choose to work attach greater importance to self-oriented motives, such as maintaining reputation, advancing career prospects, or inner convictions, and to avoidance-oriented motives, such as avoiding unpleasant situations or accumulation of unfinished work. In contrast, coefficients for well-being of others remain small and statistically insignificant across temperature levels, suggesting that prosocial considerations play only a minor role. When comparing across attendance types, broadly similar patterns emerge for both virtual and on-site SP, though the associations for self-related motives and avoidance of unpleasant situations (where visibility and social expectations are more pronounced) tend to be somewhat stronger for on-site SP, whereas the avoidance of accumulation of unfinished work seems to be more important for virtual SP.

**Table 4:** Regression analyses of work motivations on body temperature by attendance mode (total, remote work, on-site work).

	Total	Remote work	On-site work
**Inner convictions and principles**			
37.5°C	0.047	0.103	0.042
	[-0.029, 0.123]	[-0.028, 0.233]	[-0.053, 0.137]
38.3°C	**0.140**	0.105	**0.232**
	[0.036, 0.244]	[-0.054, 0.265]	[0.089, 0.376]
Mean of motivation variable	4.289	4.257	4.309
N	2056	778	1278

**What colleagues or supervisors think of me**			
37.5°C	0.048	0.095	0.028
	[-0.053, 0.148]	[-0.070, 0.260]	[-0.102, 0.158]
38.3°C	0.127	0.116	0.170
	[-0.010, 0.265]	[-0.085, 0.318]	[-0.025, 0.336]
Mean of motivation variable	2.490	2.478	2.496
N	2057	778	1279

**Reputation in the company**			
37.5°C	0.098	0.095	0.114
	[-0.004, 0.200]	[-0.073, 0.263]	[-0.018, 0.246]
38.3°C	**0.204**	0.209	0.223
	[0.063, 0.344]	[0.004, 0.414]	[0.023, 0.423]
Mean of motivation variable	2.626	2.607	2.638
N	2047	776	1271

**Professional success and career**			
37.5°C	0.086	0.111	0.055
	[-0.011, 0.183]	[-0.051, 0.273]	[-0.068, 0.178]
38.3°C	**0.323**	**0.266**	**0.361**
	[0.191, 0.456]	[0.069, 0.463]	[0.175, 0.548]
Mean of motivation variable	2.322	2.393	2.280
N	2047	777	1270

**Avoidance of unpleasantness**			
37.5°C	0.114	0.224	0.049
	[0.005, 0.221]	[0.039, 0.409]	[-0.087, 0.184]
38.3°C	**0.334**	0.257	**0.477**
	[0.196, 0.492]	[0.031, 0.483]	[0.272, 0.681]
Mean of motivation variable	2.680	2.676	2.682
N	2047	772	1275

**Well-being of others**			
37.5°C	−0.017	0.004	−0.045
	[-0.113, 0.078]	[-0.162, 0.154]	[-0.168, 0.077]
38.3°C	0.127	0.153	0.068
	[-0.003, 0.257]	[-0.040, 0.346]	[-0.115, 0.251]
Mean of motivation variable	3.728	3.797	3.686
N	2030	774	1256

**Accumulation of unfinished work**			
37.5°C	0.101	**0.256**	0.010
	[-0.007, 0.209]	[0.079, 0.433]	[-0.130, 0.149]
38.3°C	**0.367**	**0.461**	**0.330**
	[0.219, 0.515]	[0.245, 0.677]	[0.119, 0.541]
Mean of motivation variable	3.284	3.356	3.239
N	2030	773	1257

*Note:* Values in brackets represent 95% confidence intervals; Bold values indicate statistically significant coefficients (p < 0.05).

Motives for SP are largely similar between men and women (see Table A9 and A10 in the Appendix), though some differences emerge. In contrast to women, the avoidance of unpleasantness is not significantly associated with virtual SP decision at higher temperatures for men. Inner convictions and principles, on the other hand, are significantly correlated with higher temperatures for men, only. The accumulation of unfinished work is associated with virtual SP among men at increased temperatures, whereas for women it is associated with on-site SP.

## 5 Discussion

Our results are largely consistent with the conceptual framework outlined in the introduction which emphasizes the interplay between health severity, situational boundary conditions, and the expanding role of working from home in attendance decisions. The findings highlight that the severity of illness is the central determinant of attendance behavior, confirming Steers and Rhodes (1978) classical proposition that ill-health functions as the “central gatekeeper” of attendance decisions. The probability of working while ill decreases sharply with increasing symptom severity and body temperature. Compared to the severity of illness, the impact of the other situational boundary conditions is much lower. For instance, indications that the illness is infectious decreased the likelihood of choosing on-site SP by only about three percentage points (without a remote work option) or two percentage points (with a remote work option). This suggests that even when facing infectious symptoms, many employees would still attend work, thereby increasing the risk of contagion and negative effects within the organization. The findings also confirm that supervisors can exert attendance pressure, leading to increased on-site SP. Our results further demonstrate that the social dynamics of the work team influence individuals' attendance decisions. Higher team cohesion and the effects on colleagues' additional workload make it more likely that individuals decide to work despite illness. Although previous research has identified high personal workload as a predictor of SP, we found only weak evidence of a situational impact of this dimension on attendance decisions. Nevertheless, a higher situational workload was related to an increased probability of deciding to engage in virtual SP. Our vignette scenario considered the actual workload relative to the respondents’ general workload. Therefore, one possible explanation for the diverging results is that employees with a high workload may have a constantly higher tendency to work despite illness, but that the impact of varying situational workload intensity on attendance decisions is weak.

The analyses also confirm a small effect of weather conditions on attendance decisions, though only for those with a remote work option. We find that good weather slightly increases the probability of choosing on-site SP, while bad weather slightly increases the probability to choose SA. These results align with the assumption that poor weather conditions decrease motivation to work when individuals experience health issues or avoid commuting in poor weather to prevent further deterioration of their health. However, effects of the weekday, such as the Monday effect (where SA is more often chosen at the beginning of the week), were not confirmed. As Barmby (2002) pointed out, the observed higher SA rates on Mondays in observational data may also result from a censoring effect, such that illnesses beginning during the weekend are reported on Mondays (Barmby, 2002).

Although illness severity emerges as the strongest predictor of attendance decisions, a non-negligible proportion of employees still choose SP even under conditions of high body temperature. Therefore, the results confirm that SP is a common phenomenon in which employees work despite clear signs of illness, rather than only covering situations where individuals work with minor health complaints. Even in vignette scenarios where respondents face a high body temperature of 38.3°C, the mean probability of working either on-site or – when possible – remotely reaches 30 percent. Gender-specific analyses indicate that men are more likely than women to engage in SP at elevated body temperatures, showing higher tendencies for virtual SP if they have the possibility of working remotely, and higher tendencies to work on-site if they do not. Some scholars argue that SP could be functional with respect to well-being and productivity for workers with specific health conditions, such as chronic musculoskeletal disorders or long-term mental health problems (Biron et al., 2022; Holland & Collins, 2018; Sanderson & Cocker, 2013). While this may be true, our vignettes focus on acute cold symptoms with varying degrees of severity. Contrary to long-term conditions mentioned above, the typical advice for individuals experiencing such acute symptoms accompanied by a high body temperature would be to rest and recover. Hence, while working under such conditions may temporarily prevent productivity and performance loss in the short term, it may carry a risk of adverse health effects and energy depletion in the long run (Chou & Mach, 2021). Moreover, this persistence of work attendance despite severe symptoms underscores that health status alone does not fully explain attendance behavior and that situational and personal factors interact in complex ways.

Our results show that the overall probability of working while ill – irrespective of symptom severity – is higher when employees have a remote work option. In particular, compared to minor health complaints, the choice of virtual SP increases under conditions of mild or moderate symptom severity (i.e., a body temperature of 37.5°C). Therefore, options to work remotely seem to raise the threshold of illness severity that prompts workers to rest and take sick leave as previously suggested by Fiorini (2024). The availability of remote work also appears to reduce the relevance of situational boundary conditions for attendance decisions, as indicated by the lower explanatory power of these factors when telework is available. This suggests that remote work introduces a more gradual, less binary decision frame: when working remotely is possible, employees can flexibly adjust their work intensity instead of facing a strict “work or stay home” dilemma. However, this does not mean that contextual boundary conditions become irrelevant. Rather, remote work changes how and when such factors matter. In the absence of these options, contextual factors continue to structure attendance decisions, particularly under mild or moderately severe illness.

Under normal or slightly elevated body temperature attendance pressure, the perceived burden on colleagues, weather conditions and infectiousness significantly shape attendance decisions. Attendance pressure, good weather and team workload increase the likelihood of SP, whereas infectiousness reduces it. As illness severity rises, these situational influences weaken markedly, and the decision to attend work becomes less contingent on context and more on personal disposition. This interpretation is supported by the motivational analyses. As body temperature increases, respondents who still decide to work, assign higher importance to self-oriented motives related to career, reputation, and avoidance of unpleasant consequences – a pattern that is homogeneous across genders. Inner convictions also become more relevant under high severity, particularly for on-site SP decisions. In contrast, intrinsic or prosocial motives such as concern for others remain largely unaffected. The accumulation of unfinished work, however, is more strongly associated with virtual SP at higher temperatures, suggesting that virtual SP may be chosen as a strategy to prevent workload spill-over and avoid resource loss cycles (Lu & Cooper, 2022). This pattern implies that when illness severity is high, situational factors become less relevant and the decision to attend work becomes increasingly driven by enduring motivational dispositions.

Taken together, the results align with conceptual models of attendance behavior that view the decision process as hierarchical or sequential (Whysall et al., 2023). Under mild or moderate illness, employees weigh situational and contextual aspects as part of a deliberative decision process, balancing health, workload, signals of supervisors and team-related consequences. As illness severity increases, this evaluative phase tends to be skipped, and the decision is instead guided by habitual, dispositional, or identity-related drivers. The observed pattern thus supports a dual-level model in which situational boundary conditions govern attendance under ambiguity, while personal motives dominate in cases of severe impairment.

### 5.1 Limitations

Several limitations should be acknowledged. First, the data are based on hypothetical vignette scenarios. While allowing for causal inference and control over situational factors, they may not fully capture the complexity of real-world attendance behavior. Respondents’ stated intentions could differ from their actual behavior when facing real illness episodes and organizational constraints. In real-world settings, attendance decisions may be influenced by additional consequences and pressures that are difficult to reproduce fully in hypothetical scenarios. Accordingly, the estimated effects should be interpreted as effects on stated attendance decisions rather than direct estimates of actual workplace behavior. Therefore, the generalizability of vignette decisions to real-life behavior could be limited (Petzold & Wolbring, 2019). Second, the factorial survey design necessarily simplified the context of illness and work, focusing on a limited set of situational dimensions and cannot account for all relevant factors such as organizational culture, leadership style, or household and care responsibilities. Relatedly, the vignettes focus on acute, short-term illness episodes resembling common colds or flu-like symptoms, chosen because such conditions are widely relatable and typically trigger short-term attendance decisions. However, the findings may not generalize to chronic, mental, or episodic health conditions, where symptom duration, perceived controllability, work adaptations and social expectations could differ substantially.

### 5.2 Conclusions and implications

Overall, this study contributes to a more nuanced understanding of SP by demonstrating how illness severity, situational constraints, and remote work opportunities jointly shape attendance behavior. By simulating attendance decisions in a factorial survey experiment, it extends classical attendance models to contemporary work contexts and highlights that the decision to work while sick is not solely health driven but reflects a dynamic interplay of contextual boundary conditions and personal motives. The findings underscore the relevance of illness severity as a central gatekeeper of attendance behavior, while showing that contextual factors matter most under conditions of mild or moderate illness, where ambiguity is highest, and that personal motives dominate decisions under severe illness.

Remote work may reduce SA in some situations, yet it also appears to shift recovery thresholds and normalize virtual SP. These dynamics highlight an emerging tension between flexibility and adequate recovery with implications for occupational health and public health. Future research should examine whether these mechanisms operate similarly for chronic and mental health conditions and how organizational norms shape the long-term health consequences of both virtual and on-site SP.

## CRediT authorship contribution statement

**Joachim Gerich:** Writing – review & editing, Writing – original draft, Methodology, Investigation, Conceptualization. **Natalie Herdegen:** Writing – review & editing, Methodology, Investigation, Conceptualization. **Andreas Koch:** Writing – review & editing, Methodology, Investigation, Conceptualization. **Martin Kroczek:** Writing – review & editing, Software, Methodology, Investigation, Formal analysis, Data curation, Conceptualization. **Philipp Kugler:** Writing – review & editing, Software, Methodology, Investigation, Formal analysis, Data curation, Conceptualization. **Sophie-Charlotte Meyer:** Writing – review & editing, Writing – original draft, Supervision, Project administration, Methodology, Investigation, Conceptualization. **Mella Perleberg:** Writing – review & editing, Methodology, Investigation, Conceptualization. **Jochen Späth:** Writing – review & editing, Methodology, Investigation, Conceptualization.

## Research transparency statement

The study has been preregistered (https://doi.org/10.1257/rct.14193-1.0). To analyze the data, we used Stata. The analysis code can be made available upon request and will be shared publicly upon publication.

## Ethical approval statement

The factorial survey experiment was approved by the Ethics Committee of the Federal Institute for Occupational Safety and Health on 30 April 2024.

## Funding statement

This research received no specific grant from any funding agency in the public, commercial, or not-for-profit sectors.

## Declaration of competing interests

The authors declare that they have no known competing financial interests or personal relationships that could have appeared to influence the work reported in this paper.

## Data Availability

The data used in this study originate from a follow-up factorial survey experiment to the BIBB/BAuA Employment Survey 2024. The factorial survey data are available from the Federal Institute for Occupational Safety and Health (BAuA). Due to data protection regulations, access requires use via an on-site guest researcher workstation. Applications can be submitted to the BAuA Research Data Centre (Forschungsdaten@baua.bund.de).

## Footnotes

1 The vignettes were otherwise identical to those used in the main experiment group. Therefore, we pool respondents from both groups in the analysis. Results from the information experiment are not focused in this paper.

2 As a robustness check, we additionally adjusted our main specification for educational attainment, using indicators for whether respondents hold a vocational or academic degree, as education may affect both vignette interpretation and sickness-related attendance behavior. The inclusion of these controls does not materially alter our main results. The corresponding estimates are presented in Table A11 in the Appendix.

3 Interaction effects are interpreted only when at least one contrast between body temperature levels is statistically significant (p < 0.05).
