# Supplementary material for: The situational contingency of virtual and on-site workplace attendance decisions in Germany: a vignette-based approach
Source: SSM Popul Health. 2026 Sep 28;36:101979. doi: 10.1016/j.ssmph.2026.101979 (PMC13657242; doi:10.1016/j.ssmph.2026.101979)
Supplement: Supplementary file 1 [file mmc1.docx]

**Appendix**

**Table A1**: Wording of the vignette dimensions

| Dimension | Factor levels |
| --- | --- |
| Weekday | You wake up on a (1) Monday (2) Tuesday (3) Wednesday (4) Thursday (5) Friday  morning. |
| Weather | (1) It is bright outside, and the sun is shining into your bedroom. (2) It is dark outside, and it is raining heavily. |
| Cold symptoms | As you get up, you realize that you’re not feeling well. (1) You feel weak and exhausted. (2) You feel weak and exhausted, and you have a mild headache. (3) You feel weak and exhausted, and you have a severe headache. |
| Body temperature | Your body temperature is (1) normal. (2) slightly elevated (37,5°). (3) significantly elevated (38,3°). |
| Infectiousness | In the past few days,  (1) you have been in contact with a person with similar symptoms. (2) you have not been in contact with anyone showing similar symptoms. |
| Attendance pressure | Your supervisor has recently… (1) emphasized that taking sick leave due to illness is completely acceptable. (2) commented negatively on the high number of sick leaves in recent months. (3) expressed scepticisms about whether employees who have called in sick are actually unable to work. |
| Team cohesion | You are currently working… (1) with people you enjoy working with. (2) with people you don’t particularly enjoy working with. |
| Personal workload | Your personal workload is currently… (1) higher than usual. (2) neither higher nor lower than usual. (3) lower than usual. |
| Team workload | You expect that your colleagues will face… (1) significantly more work (2) only a little additional work if you don’t show up. |

**Table A2:** Summary statistics of vignette characteristics in the analysis sample

|  | Mean | SD | N (Vignettes) |
| --- | --- | --- | --- |
| **Day** |  |  |  |
| Monday | 0.196 | 0.397 | 35,730 |
| Tuesday | 0.198 | 0.398 | 35,730 |
| Wednesday | 0.204 | 0.403 | 35,730 |
| Thursday | 0.198 | 0.398 | 35,730 |
| Friday | 0.204 | 0.403 | 35,730 |
| **Weather** |  |  |  |
| Good weather | 0.504 | 0.500 | 35,730 |
| **Cold symptoms** |  |  |  |
| Weak symptoms | 0.326 | 0.469 | 35,730 |
| Medium symptoms | 0.332 | 0.471 | 35,730 |
| Strong symptoms | 0.342 | 0.474 | 35,730 |
| **Body temperature** |  |  |  |
| Normal temperature | 0.326 | 0.469 | 35,730 |
| 37.5 °C | 0.332 | 0.471 | 35,730 |
| 38.3°C | 0.342 | 0.474 | 35,730 |
| **Infectiousness** |  |  |  |
| Infectious | 0.506 | 0.500 | 35,730 |
| **Pressure** |  |  |  |
| No pressure | 0.332 | 0.471 | 35,730 |
| Low pressure | 0.336 | 0.472 | 35,730 |
| High pressure | 0.332 | 0.471 | 35,730 |
| **Team cohesion** |  |  |  |
| Like team | 0.486 | 0.500 | 35,730 |
| **Workload** |  |  |  |
| High workload | 0.324 | 0.468 | 35,730 |
| Normal workload | 0.334 | 0.472 | 35,730 |
| Low workload | 0.342 | 0.474 | 35,730 |
| **Teamload** |  |  |  |
| Colleagues affected | 0.496 | 0.500 | 35,730 |

*Source*: Factorial survey on sickness presenteeism conducted as a follow-up to the BIBB/BAuA Employment Survey 2024.

**Table A3:** Relative frequency of different choices, women

|  | Respondents without remote work option |  | Respondents with remote work option | | |  | Working without differentiation |  | Working or staying at home (remote work treated missing) |  | On-site Work^1^ |
| --- | --- | --- | --- | --- | --- | --- | --- | --- | --- | --- | --- |
|  | Working |  | Not working | Remote work | On-site work |  | (remote or on-site)^1^ |  |  |  |  |
|  | (1) |  | (2) | (3) | (4) |  | (5) |  | (6) |  | (7) |
| **Body temperature** | | | | | | | | | | | |
| Normal | 0.825 |  | 0.146 | 0.421 | 0.433 |  | 0.840 |  | 0.794 |  | 0.616 |
| 37.5 °C | 0.631 |  | 0.260 | 0.477 | 0.263 |  | 0.689 |  | 0.583 |  | 0.435 |
| 38.3°C | 0.210 |  | 0.667 | 0.260 | 0.073 |  | 0.275 |  | 0.159 |  | 0.137 |
| N | 6,730 |  | 7,660 | 7,660 | 7,660 |  | 14,390 |  | 11,444 |  | 14,390 |

*Note:* ^1^ total sample without differentiation whether working remotely is possible or not. *Source*: Factorial survey on sickness presenteeism conducted as a follow-up to the BIBB/BAuA Employment Survey 2024.

**Table A4:** Relative frequency of different choices, men

|  | Respondents without remote work option |  | Respondents with remote work option | | |  | Working without differentiation |  | Working or staying at home (remote work treated missing) |  | On-site work^1^ |
| --- | --- | --- | --- | --- | --- | --- | --- | --- | --- | --- | --- |
|  | Working |  | Not working | Remote work | On-site work |  | (remote or on-site)^1^ |  |  |  |  |
|  | (1) |  | (2) | (3) | (4) |  | (5) |  | (6) |  | (7) |
| **Body temperature** | | | | | | | | | | | |
| Normal | 0.777 |  | 0.161 | 0.467 | 0.371 |  | 0.815 |  | 0.741 |  | 0.529 |
| 37.5 °C | 0.624 |  | 0.256 | 0.522 | 0.222 |  | 0.697 |  | 0.556 |  | 0.379 |
| 38.3°C | 0.259 |  | 0.647 | 0.288 | 0.065 |  | 0.316 |  | 0.170 |  | 0.140 |
| N | 8,140 |  | 12,790 | 12,790 | 12,790 |  | 20,930 |  | 15,507 |  | 20,930 |

*Note:* ^1^ total sample without differentiation whether working remotely is possible or not. *Source*: Factorial survey on sickness presenteeism conducted as a follow-up to the BIBB/BAuA Employment Survey 2024.

**Table A5:** Average Marginal Effects (AMEs) of vignette characteristics on on-site work at different levels of body temperature among respondents without a remote work option

|  | Respondents without remote work option | | |  | Test of equality of effects at different temperature levels, p-values | | |
| --- | --- | --- | --- | --- | --- | --- | --- |
|  | Work on-site | | |  |  |  |  |
|  | Normal temp. | 37.5°C | 38.3°C |  | (2)-(1) | (3)-(1) | (3)-(2) |
|  | (1) | (2) | (3) |  |  |  |  |
| **Day** |  |  |  |  |  |  |  |
| Monday (base) |  |  |  |  |  |  |  |
| Tuesday | -0.006 | -0.006 | -0.016 |  | 0.977 | 0.605 | 0.620 |
|  | [-0.033, 0.021] | [-0.032, 0.019] | [-0.042, 0.010] |  | [-0.038, 0.037] | [-0.046, 0.027] | [-0.045, 0.027] |
| Wednesday | -0.004 | 0.006 | -0.011 |  | 0.624 | 0.704 | 0.342 |
|  | [-0.032, 0.024] | [-0.020, 0.031] | [-0.034, 0.013] |  | [-0.028, 0.047] | [-0.041, 0.028] | [-0.049, 0.017] |
| Thursday | -0.001 | 0.012 | -0.005 |  | 0.541 | 0.820 | 0.359 |
|  | [-0.029, 0.027] | [-0.015, 0.039] | [-0.030, 0.020] |  | [-0.028, 0.053] | [-0.040, 0.032] | [-0.053, 0.019] |
| Friday | 0.017 | 0.012 | 0.001 |  | 0.776 | 0.361 | 0.529 |
|  | [-0.009, 0.042] | [-0.013, 0.037] | [-0.022, 0.024] |  | [-0.041, 0.030] | [-0.050, 0.018] | [-.0.44, 0.023] |
| **Weather** |  |  |  |  |  |  |  |
| Good weather (base) |  |  |  |  |  |  |  |
| Rainy weather | -0.005 | 0.003 | 0.005 |  | 0.566 | 0.449 | 0.904 |
|  | [-0.023, 0.014] | [-0.014, 0.020] | [-0.010, 0.019] |  | [-0.019, 0.034] | [-0.015, 0.033] | [-0.021, 0.024] |
| **Cold symptoms** |  |  |  |  |  |  |  |
| Weak symptoms (base) |  |  |  |  |  |  |  |
| Medium symptoms | **-0.042** | -0.008 | 0.010 |  | **0.001** | **0.000** | 0.867 |
|  | [-0.063, -0.020] | [-0.011, 0.027] | [-0.008, 0.029] |  | [0.021, 0.079] | [0.024, 0.080] | [-0.025, 0.029] |
| Strong symptoms | **-0.201** | **-0.124** | **-0.032** |  | **0.000** | **0.000** | **0.000** |
|  | [-0.227, -0.176] | [-0.148, -0.101] | [-0.051, -0.013] |  | [0.046, 0.108] | [0.140, 0.199] | [0.063, 0.122] |
| **Infectiousness** |  |  |  |  |  |  |  |
| Infectious (base) |  |  |  |  |  |  |  |
| Not infectious | **0.025** | **0.048** | 0.006 |  | 0.067 | 0.101 | **0.000** |
|  | [0.008, 0.041] | [0.030, 0.065] | [-0.010, 0.022] |  | [-0.002, 0.047] | [-0.042, 0.004] | [-0.064, -0.019] |
| **Pressure** |  |  |  |  |  |  |  |
| No pressure (base) |  |  |  |  |  |  |  |
| Low pressure | 0.007 | **0.039** | -0.001 |  | **0.041** | 0.586 | **0.007** |
|  | [-0.014, 0.028] | [0.017, 0.060] | [-0.020, 0.019] |  | [0.001, 0.062] | [-0.036, 0.020] | [-0.068, -0.011] |
| High pressure | 0.003 | **0.036** | 0.013 |  | **0.043** | 0.502 | 0.131 |
|  | [-0.018, 0.025] | [0.013, 0.058] | [-0.006, 0.032] |  | [0.001, 0.064] | [-0.018, 0.038] | [-0.052, 0.007] |
| **Team cohesion** |  |  |  |  |  |  |  |
| Like team (base) |  |  |  |  |  |  |  |
| Not like team | -0.020 | -0.011 | -0.010 |  | 0.474 | 0.400 | 0.936 |
|  | [-0.037, -0.003] | [-0.028, 0.006] | [-0.026, 0.005] |  | [-0.015, 0.032] | [-0.013, 0.031] | [-0.021, 0.023] |
| **Workload** |  |  |  |  |  |  |  |
| High workload (base) |  |  |  |  |  |  |  |
| Normal workload | 0.002 | 0.007 | -0.008 |  | 0.743 | 0.454 | 0.270 |
|  | [-0.019, 0.024] | [-0.013, 0.028] | [-0.028, 0.011] |  | [-0.025, 0.035] | [-0.039, 0.018] | [-0.044, 0.012] |
| Low workload | 0.001 | -0.007 | -0.012 |  | 0.556 | 0.340 | 0.775 |
|  | [-0.019, 0.022] | [-0.029, 0.015] | [-0.031, 0.007] |  | [-0.038, 0.021] | [-0.040, 0.014] | [-0.033, 0.025] |
| **Teamload** |  |  |  |  |  |  |  |
| Colleagues affected (base) |  |  |  |  |  |  |  |
| Colleagues not affected | **-0.048** | **-0.060** | **-0.037** |  | 0.331 | 0.323 | **0.049** |
|  | [-0.065, -0.032] | [-0.078, -0.042] | [-0.053, -0.022] |  | [-0.035, 0.012] | [-0.011, 0.032] | [0.000, 0.045] |
| Control for potential participation in other experiment | ✓ |  |  |  |  |  |  |
| N | 14,870 |  |  |  |  |  |  |
| Overall Pseudo R² | 0.433 |  |  |  |  |  |  |

*Note:* In order to account for the fact that certain groups of respondents were less likely to participate in the main experiment due to their allocation to another experiment, membership of a group that potentially participated in another experiment is included as a control variable in the estimation models; Average marginal effects are based on a binary logistic regression model including interactions between body temperature and all vignette dimensions; Values in brackets represent 95% confidence intervals; Bold values indicate statistically significant coefficients/tests of equality (p<0.05); Standard errors clustered at the respondent level. *Source*: Factorial survey on sickness presenteeism conducted as a follow-up to the BIBB/BAuA Employment Survey 2024.

**Table A6:** Average Marginal Effects (AMEs) of vignette characteristics on remote work at different levels of body temperature among respondents with a remote work option

|  | Respondents with remote work option | | |  | Test of equality of effects at different temperature levels, p-values | | |
| --- | --- | --- | --- | --- | --- | --- | --- |
|  | Working remotely | | |  |  |  |  |
|  | Normal temp. | 37.5°C | 38.3°C |  | (2)-(1) | (3)-(1) | (3)-(2) |
|  | (1) | (2) | (3) |  |  |  |  |
| **Day** |  |  |  |  |  |  |  |
| Monday (base) |  |  |  |  |  |  |  |
| Tuesday | -0.012 | -0.003 | 0.023 |  | 0.616 | 0.056 | 0.115 |
|  | [-0.038, 0.015] | [-0.025, 0.019] | [-0.001, 0.046] |  | [-0.025, 0.043] | [-0.001, 0.070] | [-0.006, 0.058] |
| Wednesday | 0.002 | -0.006 | 0.009 |  | 0.648 | 0.719 | 0.353 |
|  | [-0.025, 0.029] | [-0.027, 0.016] | [-0.013, 0.031] |  | [-0.043, 0.027] | [-0.029, 0.042] | [-0.016, 0.045] |
| Thursday | 0.010 | 0.007 | 0.008 |  | 0.857 | 0.928 | 0.922 |
|  | [-0.019, 0.038] | [-0.015, 0.028] | [-0.016, 0.032] |  | [-0.039,0.033] | [-0.039,0.035] | [-0.031, 0.034] |
| Friday | 0.015 | 0.009 | 0.000 |  | 0.716 | 0.372 | 0.559 |
|  | [-0.011, 0.042] | [-0.012, 0.030] | [-0.021, 0.021] |  | [-0.040,0.027] | [-0.049, 0.018] | [-0.040, 0,021] |
| **Weather** |  |  |  |  |  |  |  |
| Good weather (base) |  |  |  |  |  |  |  |
| Rainy weather | 0.002 | -0.002 | -0.006 |  | 0.719 | 0.470 | 0.687 |
|  | [-0.015, 0.019] | [-0.016, 0.012] | [-0.021, 0.009] |  | [-0.027, 0.019] | [-0.031, 0.014] | [-0.025, 0.016] |
| **Cold symptoms** |  |  |  |  |  |  |  |
| Weak symptoms (base) |  |  |  |  |  |  |  |
| Medium symptoms | -0.010 | -0.004 | **0.031** |  | 0.656 | **0.005** | **0.005** |
|  | [-.032, 0.012] | [-0.020, 0.012] | [0.012, 0.050] |  | [-0.021, 0.033] | [0.012, 0.070] | [0.011, 0.059] |
| Strong symptoms | **-0.074** | **-0.087** | **-0.030** |  | 0.350 | **0.003** | **0.000** |
|  | [-0.099, -0.049] | [-0.011, -0.068] | [-0.048, -0.012] |  | [-0.042, 0.015] | [0.015, 0.073] | [0.033, 0.082] |
| **Infectiousness** |  |  |  |  |  |  |  |
| Infectious (base) |  |  |  |  |  |  |  |
| Not infectious | **-0.025** | 0.002 | 0.006 |  | **0.015** | **0.004** | 0.632 |
|  | [-0.041, -0.009] | [-0.012, 0.016] | [-0.008, 0.021] |  | [0.005, 0.048] | [0.001, 0.053] | [-0.015, 0.025] |
| **Pressure** |  |  |  |  |  |  |  |
| No pressure (base) |  |  |  |  |  |  |  |
| Low pressure | -0.018 | 0.005 | **-0.029** |  | 0.099 | **0.001** | **0.045** |
|  | [-0.038, 0.003] | [-0.011, 0.022] | [0.012, 0.047] |  | [-0.004, 0.050] | [0.020, 0.074] | [0.000, 0.048] |
| High pressure | **-0.035** | 0.007 | 0.013 |  | **0.005** | **0.001** | 0.629 |
|  | [-0.056, -0.013] | [-0.011, 0.024] | [-0.005, 0.030] |  | [0.013, 0.070] | [0.020, 0.075] | [-0.018, 0.030] |
| **Team cohesion** |  |  |  |  |  |  |  |
| Like team (base) |  |  |  |  |  |  |  |
| Not like team | -0.009 | 0.003 | **-0.019** |  | 0.295 | 0.357 | **0.029** |
|  | [-0.026, 0.008] | [-0.011, 0.016] | [-0.033, -0.005] |  | [-0.010, 0.033] | [-0.032, 0.012] | [-0.041, -0.002] |
| **Workload** |  |  |  |  |  |  |  |
| High workload (base) |  |  |  |  |  |  |  |
| Normal workload | -0.018 | -0.001 | -0.022 |  | 0.215 | 0.811 | 0.082 |
|  | [-0.040, 0.003] | [-0.018, 0.016] | [-0.040, -0.004] |  | [-0.010, 0.046] | [-0.031, 0.024] | [-0.045, 0.003] |
| Low workload | -0.021 | -0.013 | -0.023 |  | 0.566 | 0.877 | 0.410 |
|  | [-0.042, -0.000] | [-0.030, 0.004] | [-0.041, -0.005] |  | [-0.019, 0.035] | [-0.029, 0.025] | [-0.034, 0.014] |
| **Teamload** |  |  |  |  |  |  |  |
| Colleagues affected (base) |  |  |  |  |  |  |  |
| Colleagues not affected | **-0.024** | -0.017 | **-0.031** |  | 0.525 | 0.523 | 0.169 |
|  | [-0.041, -0.007] | [-0.031, -0.002] | [-0.046, -0.016] |  | [-0.015, 0.029] | [-0.029, 0.015] | [-0.034, 0.006] |
|  |  |  |  |  |  |  |  |
| Control for potential participation in other experiment | ✓ |  |  |  |  |  |  |
| N | 20,450 |  |  |  |  |  |  |
| Overall Pseudo R² | 0.408 |  |  |  |  |  |  |

*Note:* In order to account for the fact that certain groups of respondents were less likely to participate in the main experiment due to their allocation to another experiment, membership of a group that potentially participated in another experiment is included as a control variable in the estimation models; Average marginal effects in Tables A6-A8 are based on the same multinomial logistic regression model including interactions between body temperature and all vignette dimensions with results reported separately by outcome category; Values in brackets represent 95% confidence intervals; Bold values indicate statistically significant coefficients/tests of equality (p<0.05); Standard errors clustered at the respondent level. *Source*: Factorial survey on sickness presenteeism conducted as a follow-up to the BIBB/BAuA Employment Survey 2024.

**Table A7:** Average Marginal Effects (AMEs) of vignette characteristics on on-site work at different levels of body temperature among respondents with a remote work option

|  | Respondents with remote work option | | |  | Test of equality of effects at different temperature levels, p-values | | |
| --- | --- | --- | --- | --- | --- | --- | --- |
|  | Working on-site | | |  |  |  |  |
|  | Normal temp. | 37.5°C | 38.3°C |  | (2)-(1) | (3)-(1) | (3)-(2) |
|  | (1) | (2) | (3) |  |  |  |  |
| **Day** |  |  |  |  |  |  |  |
| Monday (base) |  |  |  |  |  |  |  |
| Tuesday | -0.011 | -0.005 | -0.008 |  | 0.688 | 0.867 | 0.760 |
|  | [-0.033, 0.012] | [-0.019, 0.009] | [-0.024, 0.007] |  | [-0.021, 0.032] | [-0.025, 0.030] | [-0.024, 0.017] |
| Wednesday | 0.007 | 0.001 | -0.012 |  | 0.647 | 0.146 | 0.179 |
|  | [-0.015, 0.030] | [-0.013, 0.015] | [-0.026, 0.002] |  | [-0.034, 0.021] | [-0.047, 0.007] | [-0.033, 0.006] |
| Thursday | -0.016 | 0.000 | 0.000 |  | 0.247 | 0.265 | 0.989 |
|  | [-0.040, 0.008] | [-0.013, 0.014] | [-0.016, 0.016] |  | [-0.011, 0.044] | [-0.012, 0.044] | [-0.021,0.021] |
| Friday | -0.013 | -0.003 | -0.001 |  | 0.449 | 0.378 | 0.854 |
|  | [-0.035, 0.010] | [-0.016, 0.011] | [-0.016, 0.014] |  | [-0.016, 0.037] | [-0.015, 0.039] | [-0.018, 0.022] |
| **Weather** |  |  |  |  |  |  |  |
| Good weather (base) |  |  |  |  |  |  |  |
| Rainy weather | **-0.020** | -0.009 | 0.001 |  | 0.170 | **0.012** | 0.130 |
|  | [-0.035, -0.006] | [-0.017, 0.000] | [-0.008, 0.010] |  | [-0.005, 0.028] | [0.005, 0.038] | [-0.003, 0.002] |
| **Cold symptoms** |  |  |  |  |  |  |  |
| Weak symptoms (base) |  |  |  |  |  |  |  |
| Medium symptoms | -0.009 | 0.006 | -0.007 |  | 0.181 | 0.817 | 0.124 |
|  | [-0.030, 0.011] | [-0.005, 0.016] | [-0.019, 0.006] |  | [-0.007, 0.037] | [-0.020, 0.025] | [-0.028, 0.003] |
| Strong symptoms | **-0.115** | **-0.040** | **-0.015** |  | **0.000** | **0.000** | **0.003** |
|  | [-0.136, -0.094] | [-0.053, -0.028] | [-0.026, -0.004] |  | [0.052, 0.097] | [0.076, 0.124] | [0.009, 0.042] |
| **Infectiousness** |  |  |  |  |  |  |  |
| Infectious (base) |  |  |  |  |  |  |  |
| Not infectious | **0.038** | **0.021** | 0.004 |  | **0.043** | **0.000** | **0.010** |
|  | [0.024, 0.051] | [0.012, 0.030] | [-0.005, 0.014] |  | [-0.032, -0.001] | [-0.050, -0.017] | [-0.030, -0.004] |
| **Pressure** |  |  |  |  |  |  |  |
| No pressure (base) |  |  |  |  |  |  |  |
| Low pressure | 0.020 | 0.004 | -0.005 |  | 0.105 | **0.013** | 0.273 |
|  | [0.004, 0.037] | [-0.007, 0.014] | [-0.017, 0.007] |  | [-0.037, 0.003] | [-0.024, 0.007] | [-0.024, 0,007] |
| High pressure | **0.026** | 0.011 | 0.001 |  | 0.162 | **0.018** | 0.230 |
|  | [0.008, 0.044] | [-0.001, 0.022] | [-0.011, 0.012] |  | [-0.037, 0.006] | [-0.046, -0.004] | [-0.026, 0.006] |
| **Team cohesion** |  |  |  |  |  |  |  |
| Like team (base) |  |  |  |  |  |  |  |
| Not like team | **-0.021** | **-0.013** | -0.009 |  | 0.293 | 0.130 | 0.530 |
|  | [-0.035, -0.007] | [-0.022, -0.004] | [-0.018, 0.001] |  | [-0.007, 0.025] | [-0.004, 0.029] | [-0.009, 0.017] |
| **Workload** |  |  |  |  |  |  |  |
| High workload (base) |  |  |  |  |  |  |  |
| Normal workload | 0.015^#^ | -0.003 | -0.002 |  | 0.083 | 0.109 | 0.840 |
|  | [-0.002, 0.032] | [-0.014, 0.008] | [-0.012, 0.009] |  | [-0.040, 0.002] | [-0.038, 0.038] | [-0.014, 0.017] |
| Low workload | 0.008 | -0.002 | -0.003 |  | 0.363 | 0.319 | 0.940 |
|  | [-0.009, 0.024] | [-0,013, 0.009] | [-0.014, 0.009] |  | [-0.030, 0.011] | [-0.030, 0.010] | [-0.017, 0.015] |
| **Teamload** |  |  |  |  |  |  |  |
| Colleagues affected (base) |  |  |  |  |  |  |  |
| Colleagues not affected | -0.014 | **-0.018** | **-0.015** |  | 0.692 | 0.966 | 0.664 |
|  | [-0.028, -0.001] | [-0.027, -0.008] | [-0.024, -0.005] |  | [-0.019, 0.013] | [-0.017, 0.016] | [-0.010, 0.016] |
| Control for potential participation in other experiment | ✓ |  |  |  |  |  |  |
| N | 20,450 |  |  |  |  |  |  |
| Overall Pseudo R² | 0.408 |  |  |  |  |  |  |

*Note:* In order to account for the fact that certain groups of respondents were less likely to participate in the main experiment due to their allocation to another experiment, membership of a group that potentially participated in another experiment is included as a control variable in the estimation models; Average marginal effects in Tables A6-A8 are based on the same multinomial logistic regression model including interactions between body temperature and all vignette dimensions with results reported separately by outcome category; Values in brackets represent 95% confidence intervals; Bold values indicate statistically significant coefficients/tests of equality (p<0.05); Standard errors clustered at the respondent level. *Source*: Factorial survey on sickness presenteeism conducted as a follow-up to the BIBB/BAuA Employment Survey 2024.

**Table A8:** Average Marginal Effects (AMEs) of vignette characteristics on work absence at different levels of body temperature among respondents with a remote work option

|  | Respondents with remote work option | | |  | Test of equality of effects at different temperature levels, p-values | | |
| --- | --- | --- | --- | --- | --- | --- | --- |
|  | Not working | | |  |  |  |  |
|  | Normal temp. | 37.5°C | 38.3°C |  | (2)-(1) | (3)-(1) | (3)-(2) |
|  | (1) | (2) | (3) |  |  |  |  |
| **Day** |  |  |  |  |  |  |  |
| Monday (base) |  |  |  |  |  |  |  |
| Tuesday | 0.023 | 0.008 | -0.014 |  | 0.354 | 0.023 | 0.156 |
|  | [0.001, 0.045] | [-0.012, 0.029] | [-0.038, 0.010] |  | [-0.044, 0.016] | [-0.069, -0.005] | [-0.054, 0.009 |
| Wednesday | -0.010 | 0.005 | 0.004 |  | 0.358 | 0.393 | 0.942 |
|  | [-0.032, 0.013] | [-0.016, 0.026] | [-0.018, 0.025] |  | [-0.016, 0.045] | [-0.017, 0.044] | [-0.031, 0.029] |
| Thursday | 0.006 | -0.007 | -0.008 |  | 0.433 | 0.403 | 0.928 |
|  | [-0.018, 0.030] | [-0.028, 0.014] | [-0.033, 0.016] |  | [-0.045, 0.019] | [-0.048,0.019] | [-0.033, 0.031] |
| Friday | -0.002 | -0.006 | 0.001 |  | 0.791 | 0.847 | 0.648 |
|  | [-0.025, 0.020] | [-0.027, 0.014] | [-0.021, 0.022] |  | [-0.034, 0.026] | [-0.027, 0.034] | [-0.023, 0.037] |
| **Weather** |  |  |  |  |  |  |  |
| Good weather (base) |  |  |  |  |  |  |  |
| Rainy weather | 0.018 | 0.011 | 0.005 |  | 0.455 | 0.213 | 0.583 |
|  | [0.004, 0.032] | [-0.002, 0.023] | [-0.009, 0.020] |  | [-0.027, 0.012] | [-0.033, 0.077] | [-0.025, 0.014] |
| **Cold symptoms** |  |  |  |  |  |  |  |
| Weak symptoms (base) |  |  |  |  |  |  |  |
| Medium symptoms | 0.019 | -0.002 | -0.024 |  | 0.052 | **0.001** | 0.072 |
|  | [0.003, 0.036] | [-0.026, 0.012] | [-0.044, -0.005] |  | [-0.043, 0.000] | [-0.068, -0.019] | [-0.047, 0.002] |
| Strong symptoms | **0.188** | **0.128** | **0.045** |  | **0.000** | **0.000** | **0.000** |
|  | [0.167, 0.210] | [0.109, 0.147] | [0.027, 0.063] |  | [-0.086, -0.036] | [-0.170, -0.117] | [-0.108, -0.058] |
| **Infectiousness** |  |  |  |  |  |  |  |
| Infectious (base) |  |  |  |  |  |  |  |
| Not infectious | -0.013 | **-0.023** | -0.011 |  | 0.304 | 0.835 | 0.219 |
|  | [-0.026, 0.001] | [-0.036, -0.009] | [-0.025, 0.004] |  | [-0.029,0.009] | [-0.018, 0.022] | [-0.007, 0.032] |
| **Pressure** |  |  |  |  |  |  |  |
| No pressure (base) |  |  |  |  |  |  |  |
| Low pressure | -0.003 | -0.009 | **-0.025** |  | 0.605 | 0.077 | 0.208 |
|  | [-0.020, 0.014] | [-0.026, 0.007] | [-0.043, -0.006] |  | [-0.031, 0.018] | [-0.046, 0.002] | [-0.039, 0.009] |
| High pressure | 0.009 | -0.017 | -0.013 |  | **0.044** | 0.082 | 0.746 |
|  | [-0.009, 0.026] | [-0.034, -0.000] | [-0.031, 0.004] |  | [-0.051, -0.001] | [-0.047, 0.003] | [-0.020, 0.028] |
| **Team cohesion** |  |  |  |  |  |  |  |
| Like team (base) |  |  |  |  |  |  |  |
| Not like team | **0.030** | 0.010 | **0.028** |  | **0.042** | 0.811 | 0.073 |
|  | [0.016, 0.044] | [-0.003, 0.023] | [0.013, 0.042] |  | [-0.039, -0.001] | [-0.022, 0.017] | [-0.002, 0,037] |
| **Workload** |  |  |  |  |  |  |  |
| High workload (base) |  |  |  |  |  |  |  |
| Normal workload | 0.003 | 0.004 | 0.023 |  | 0.965 | 0.116 | 0.102 |
|  | [-0.015, 0.022] | [-0.012, 0.020] | [0.005, 0.042] |  | [-0.027, 0.026] | [-0005, 0.050] | [-0.004, 0.043] |
| Low workload | 0.013 | 0.015 | **0.026** |  | 0.895 | 0.332 | 0.386 |
|  | [-0.004, 0.031] | [-0.002, 0.032] | [0.008, 0.044] |  | [-0.023,0.026] | [-0.013, 0.037] | [-0.014, 0.035] |
| **Teamload** |  |  |  |  |  |  |  |
| Colleagues affected (base) |  |  |  |  |  |  |  |
| Colleagues not affected | **0.038** | **0.034** | **0.046** |  | 0.699 | 0.461 | 0.263 |
|  | [0.024, 0.052] | [0.020, 0.048] | [0.030, 0.061] |  | [-0.024, 0.016] | [-0.012, 0.027] | [-0.008, 0.031] |
| Control for potential participation in other experiment | ✓ |  |  |  |  |  |  |
| N | 20,450 |  |  |  |  |  |  |
| Overall Pseudo R² | 0.408 |  |  |  |  |  |  |

*Note:* In order to account for the fact that certain groups of respondents were less likely to participate in the main experiment due to their allocation to another experiment, membership of a group that potentially participated in another experiment is included as a control variable in the estimation models; Average marginal effects in Tables A6-A8 are based on the same multinomial logistic regression model including interactions between body temperature and all vignette dimensions with results reported separately by outcome category; Values in brackets represent 95% confidence intervals; Bold values indicate statistically significant coefficients/tests of equality (p<0.05); Standard errors clustered at the respondent level. *Source*: Factorial survey on sickness presenteeism conducted as a follow-up to the BIBB/BAuA Employment Survey 2024.

**Table A9:** Regression analyses of work motivations on body temperature by attendance mode (total, remote work, on-site work), women

|  | Total |  | Remote work |  | On-site work |
| --- | --- | --- | --- | --- | --- |
| **Inner convictions and principles** |  | | | | |
| 37.5°C | 0.041 |  | 0.024 |  | 0.066 |
|  | [-0.070, 0.152] |  | [-0.188, 0.235] |  | [-0.068, 0.199] |
| 38.3°C | 0.055 |  | 0.040 |  | 0.105 |
|  | [-0.102, 0.213] |  | [-0.212, 0.290] |  | [-0.110, 0.320] |
| Mean of motivation variable | 4.310 |  | 4.265 |  | 4.331 |
| N | 846 |  | 275 |  | 571 |
| **What colleagues or supervisors think of me** |  | | | | |
| 37.5°C | 0.081 |  | 0.299 |  | -0.030 |
|  | [-0.078, 0.241] |  | [-0.000, 0.598] |  | [-0.225, 0.165] |
| 38.3°C | 0.174 |  | 0.210 |  | 0.202 |
|  | [-0.053, 0.401] |  | [-0.146, 0.567] |  | [-0.112, 0.516] |
| Mean of motivation variable | 2.602 |  | 2.607 |  | 2.600 |
| N | 847 |  | 275 |  | 572 |
| **Reputation in the company** |  | | | | |
| 37.5°C | 0.048 |  | 0.211 |  | -0.017 |
|  | [-0.109, 0.204] |  | [-0.081, 0.504] |  | [-0.209, 0.174] |
| 38.3°C | 0.247 |  | 0.196 |  | 0.344 |
|  | [0.024, 0.469] |  | [-0.152, 0.544] |  | [0.037, 0.651] |
| Mean of motivation variable | 2.648 |  | 2.631 |  | 2.656 |
| N | 841 |  | 274 |  | 567 |
| **Professional success and career** |  | | | | |
| 37.5°C | -0.063 |  | 0.056 |  | -0.140 |
|  | [-0.209, 0.082] |  | [-0.225, 0.337] |  | [-0.316, 0.035] |
| 38.3°C | **0.354** |  | 0.240 |  | **0.450** |
|  | [0.147, 0.561] |  | [-0.095, 0.574] |  | [0.169, 0.732] |
| Mean of motivation variable | 2.283 |  | 2.376 |  | 2.239 |
| N | 840 |  | 274 |  | 566 |
| **Avoidance of unpleasantness** |  | | | | |
| 37.5°C | 0.133 |  | 0.290 |  | 0.050 |
|  | [-0.035, 0.302] |  | [-0.034, 0.613] |  | [-0.154, 0.254] |
| 38.3°C | **0.453** |  | 0.371 |  | **0.572** |
|  | [0.212, 0.694] |  | [-0.013, 0.755] |  | [0.241, 0.902] |
| Mean of motivation variable | 2.749 |  | 2.768 |  | 2.740 |
| N | 842 |  | 272 |  | 570 |
| **Well-being of others** |  | | | | |
| 37.5°C | 0.041 |  | -0.085 |  | 0.056 |
|  | [-0.100, 0.181] |  | [-0.360, 0.189] |  | [-0.112, 0.225] |
| 38.3°C | 0.188 |  | 0.166 |  | 0.121 |
|  | [-0.011, 0.387] |  | [-0.160, 0.492] |  | [-0.147,0.389] |
| Mean of motivation variable | 3.782 |  | 3.832 |  | 3.758 |
| N | 831 |  | 273 |  | 558 |
| **Accumulation of unfinished work** |  | | | | |
| 37.5°C | 0.047 |  | 0.147 |  | -0.006 |
|  | [-0.118, 0.212] |  | [-0.148, 0.443] |  | [-0.211, 0.199] |
| 38.3°C | **0.346** |  | 0.102 |  | **0.562** |
|  | [0.111, 0.580] |  | [-0.248, 0.451] |  | [-0.238, 0.354] |
| Mean of motivation variable | 3.382 |  | 3.489 |  | 3.330 |
| N | 829 |  | 272 |  | 557 |
| *Note:* Dependent variables on motives are measured on a scale of 1 to 5, where 1 refers to very unimportant and 5 to very important. Base category of temperature = normal temperature; Values in brackets represent 95% confidence intervals; Bold values indicate statistically significant coefficients (p<0.05); Standard errors clustered at the respondent level. *Source*: Factorial survey on sickness presenteeism conducted as a follow-up to the BIBB/BAuA Employment Survey 2024. | | | | | |

**Table A10:** Regression analyses of work motivations on body temperature by attendance mode (total, remote work, on-site work), men

|  | Total |  | Remote work |  | On-site work |
| --- | --- | --- | --- | --- | --- |
| **Inner convictions and principles** |  | | | | |
| 37.5°C | 0.056 |  | 0.155 |  | 0.025 |
|  | [-0.048, 0.160] |  | [-0.015, 0.234] |  | [-0.110, 0.160] |
| 38.3°C | **0.201** |  | 0.144 |  | **0.330** |
|  | [0.062,0.341] |  | [-0.065, 0.353] |  | [0.133, 0.526] |
| Mean of motivation variable | 4.275 |  | 4.252 |  | 4.291 |
| N | 1,210 |  | 503 |  | 707 |
| **What colleagues or supervisors think of me** |  | | | | |
| 37.5°C | 0.028 |  | -0.007 |  | 0.068 |
|  | [-0.102, 0.158] |  | [-0.209, 0.195] |  | [-0.107, 0.242] |
| 38.3°C | 0.112 |  | 0.079 |  | 0.195 |
|  | [-0.062, 0.285] |  | [-0.169, 0.326] |  | [-0.059, 0.448] |
| Mean of motivation variable | 2.411 |  | 2.408 |  | 2.413 |
| N | 1,210 |  | 503 |  | 707 |
| **Reputation in the company** |  | | | | |
| 37.5°C | 0.139 |  | 0.024 |  | 0.221 |
|  | [0.002, 0.275] |  | [-0.188, 0.236] |  | [0.037, 0.405] |
| 38.3°C | 0.190 |  | 0.215 |  | 0.155 |
|  | [0.006, 0.373] |  | [-0.045, 0.474] |  | [-0.114, 0.423] |
| Mean of motivation variable | 2.611 |  | 2.594 |  | 2.624 |
| N | 1,206 |  | 502 |  | 704 |
| **Professional success and career** |  | | | | |
| 37.5°C | **0.200** |  | 0.123 |  | **0.225** |
|  | [0.070, 0.329] |  | [-0.083, 0.328] |  | [0.054, 0.396] |
| 38.3°C | **0.315** |  | 0.256 |  | 0.316 |
|  | [0.141, 0.490] |  | [0.004, 0.508] |  | [0.066, 0.566] |
| Mean of motivation variable | 2.350 |  | 2.402 |  | 2.313 |
| N | 1,207 |  | 503 |  | 704 |
| **Avoidance of unpleasantness** |  | | | | |
| 37.5°C | 0.099 |  | 0.173 |  | 0.044 |
|  | [-0.42, 0.241] |  | [-0.059, 0.404] |  | [-0.139, 0.227] |
| 38.3°C | **0.285** |  | 0.193 |  | **0.423** |
|  | [0.096, 0.475] |  | [-0.092, 0.478] |  | [0.158, 0.689] |
| Mean of motivation variable | 2.632 |  | 2.626 |  | 2.635 |
| N | 1,205 |  | 500 |  | 705 |
| **Well-being of others** |  | | | | |
| 37.5°C | -0.048 |  | 0.059 |  | -0.136 |
|  | [-0.177, 0.081] |  | [-0.141, 0.259] |  | [-0.311, 0.039] |
| 38.3°C | 0.098 |  | 0.137 |  | 0.034 |
|  | [-0.074, 0.271] |  | [-0.109, 0.382] |  | [-0.218, 0.286] |
| Mean of motivation variable | 3.691 |  | 3.778 |  | 3.628 |
| N | 1,199 |  | 501 |  | 698 |
| **Accumulation of unfinished work** |  | | | | |
| 37.5°C | 0.153 |  | **0.373** |  | 0.022 |
|  | [0.010, 0295] |  | [0.150, 0.956] |  | [-0.170, 0.214] |
| 38.3°C | **0.395** |  | **0.659** |  | 0.192 |
|  | [0.203, 0.586] |  | [0.385, 0.984] |  | [-0.086, 0.470] |
| Mean of motivation variable | 3.216 |  | 3.283 |  | 3.167 |
| N | 1,201 |  | 501 |  | 700 |
| *Note:* Dependent variables on motives are measured on a scale of 1 to 5, where 1 refers to very unimportant and 5 to very important. Base category of temperature = normal temperature; Values in brackets represent 95% confidence intervals Bold values indicate statistically significant coefficients (p<0.05); Standard errors clustered at the respondent level; *Source*: Factorial survey on sickness presenteeism conducted as a follow-up to the BIBB/BAuA Employment Survey 2024. | | | | | |

**Table A11:** Average Marginal Effects (AMEs) of vignette characteristics on work attendance, additional control for vocational or academic qualification

|  | Respondents without remote work option |  | Respondents with remote work option | | |  | Pseudo-R² | |
| --- | --- | --- | --- | --- | --- | --- | --- | --- |
|  | On-site work |  | Not working | Remote work | On-site Work |  | without remote work option | with remote work option |
|  | (1) |  | (2) | (3) | (4) |  | (5) | (6) |
| **Day** |  |  |  |  |  |  | 0 | 0 |
| Monday (base) |  |  |  |  |  |  |  |  |
| Tuesday | -0.009 |  | 0.005 | 0.003 | -0.009 |  |  |  |
|  | [-0.024, 0.005] |  | [-0.008, 0.018] | [-0.011, 0.017] | [-0.018, 0.001] |  |  |  |
| Wednesday | -0.002 |  | 0.000 | 0.001 | -0.002 |  |  |  |
|  | [-0.017, 0.013] |  | [-0.012, 0.012] | [-0.011, 0.015] | [-0.012, 0.007] |  |  |  |
| Thursday | 0.001 |  | -0.002 | 0.006 | -0.004 |  |  |  |
|  | [-0.014, 0.017] |  | [-0.015, 0.011] | [-0.007, 0.021] | [-0.015, 0.006] |  |  |  |
| Friday | -0.008 |  | -0.003 | 0.009 | -0.006 |  |  |  |
|  | [-0.006, 0.023] |  | [-0.016, 0.009] | [-0.005, 0.022] | [-0.015, 0.004] |  |  |  |
| **Weather** |  |  |  |  |  |  | 0 | 0 |
| Good weather (base) |  |  |  |  |  |  |  |  |
| Rainy weather | 0.001 |  | **0.011** | -0.002 | **-0.009** |  |  |  |
|  | [-0.008, 0.011] |  | [0.003, 0.019] | [-0.011, 0.006] | [-0.015, -0.003] |  |  |  |
| **Cold symptoms** |  |  |  |  |  |  | 0.040 | 0.034 |
| Weak symptoms (base) |  |  |  |  |  |  |  |  |
| Medium symptoms | -0.005 |  | -0.004 | 0.005 | -0.001 |  |  |  |
|  | [-0.016, 0.006] |  | [-0.013, 0.006] | [-0.006, 0.016] | [-0.010, 0.006] |  |  |  |
| Strong symptoms | **-0.119** |  | **0.122** | **-0.064** | **-0.058** |  |  |  |
|  | [-0.133, -0.105] |  | [0.110, 0.135] | [-0.077, -0.051] | [-0.067, -0.049] |  |  |  |
| **Body temperature** |  |  |  |  |  |  | 0.326 | 0.246 |
| Normal (base) |  |  |  |  |  |  |  |  |
| 37.5 °C | **-0.153** |  | **0.086** | **0.027** | **-0.113** |  |  |  |
|  | [-0.170, -0.136] |  | [0.074, 0.099] | [0.011, 0.043] | [-0.129 -0.098] |  |  |  |
| 38.3°C | **-0.537** |  | **0.470** | **-0.188** | **-0.280** |  |  |  |
|  | [-0.560, -0.514] |  | [0.452, 0.490] | [-0.209, -0.168] | [-0.30, -0.265] |  |  |  |
| **Infectiousness** |  |  |  |  |  |  | 0.003 | 0.002 |
| Infectious (base) |  |  |  |  |  |  |  |  |
| Not infectious | **0.027** |  | **-0.014** | -0.007 | **0.021** |  |  |  |
|  | [0.017, 0.37] |  | [-0.023, -0.006] | [-0.016, 0.002] | [0.015, 0.027] |  |  |  |
| **Pressure** |  |  |  |  |  |  | 0.001 | 0.001 |
| No pressure (base) |  |  |  |  |  |  |  |  |
| Low pressure | 0.015 |  | -0.012 | 0.005 | 0.007 |  |  |  |
|  | [0.003, 0.027] |  | [-0.022, -0.002] | [-0.006, 0.016] | [-0.000, 0.014] |  |  |  |
| High pressure | **0.019** |  | -0.007 | -0.004 | **0.012** |  |  |  |
|  | [0.007, 0.031] |  | [-0.017, 0.002] | [-0.015, 0.006] | [0.004, 0.019] |  |  |  |
| **Team cohesion** |  |  |  |  |  |  | 0.001 | 0.001 |
| Like team (base) |  |  |  |  |  |  |  |  |
| Not like team | **-0.013** |  | **0.021** | -0.007 | **-0.014** |  |  |  |
|  | [-0.023, -0.003] |  | [0.013, 0.029] | [-0.016, 0.002] | [-0.020, -0.008] |  |  |  |
| **Workload** |  |  |  |  |  |  | 0 | 0 |
| High workload (base) |  |  |  |  |  |  |  |  |
| Normal workload | 0.001 |  | 0.007 | -0.010 | 0.002 |  |  |  |
|  | [-0.010, 0.013] |  | [-0.003, 0.018] | [-0.021, 0.001] | [-0.005, 0.010] |  |  |  |
|  |  |  |  |  |  |  |  |  |
| Low workload | -0.006 |  | **0.016** | **-0.017** | 0.001 |  |  |  |
|  | [-0.018, 0.006] |  | [0.006, 0.027] | [-0.028, -0.006] | [-0.007, 0.008] |  |  |  |
| **Teamload** |  |  |  |  |  |  | 0.008 | 0.004 |
| Colleagues affected (base) |  |  |  |  |  |  |  |  |
| Colleagues not affected | **-0.047** |  | **0.039** | **-0.024** | **-0.015** |  |  |  |
|  | [-0.057, -0.037] |  | [0.030, 0.048] | [-0.033, -0.014] | [-0.022, -0.009] |  |  |  |
| Control for potential participation in other experiment | ✓ |  | ✓ | ✓ | ✓ |  |  |  |
| Control for vocational/ academic qualification | ✓ |  | ✓ | ✓ | ✓ |  |  |  |
| N | 14,870 |  | 20,450 | 20,450 | 20,450 |  |  |  |
| Overall Pseudo R² |  |  |  |  |  |  | 0.426 | 0.405 |

*Note:* Average marginal effects from binary (Column 1) and multinomial (Column 2-4) multilevel regressions; Values in brackets represent 95% confidence intervals; Bold values indicate statistically significant coefficients (p<0.05); Standard errors clustered at the respondent level; *Source*: Factorial survey on sickness presenteeism conducted as a follow-up to the BIBB/BAuA Employment Survey 2024.
